# A systematic review: Virtual-reality-based techniques for human exercises and health improvement

**DOI:** 10.3389/fpubh.2023.1143947

**Published:** 2023-03-23

**Authors:** Saba Ghazanfar Ali, Xiangning Wang, Ping Li, Younhyun Jung, Lei Bi, Jinman Kim, Yuting Chen, David Dagan Feng, Nadia Magnenat Thalmann, Jihong Wang, Bin Sheng

**Affiliations:** ^1^Department of Computer Science and Engineering, Shanghai Jiao Tong University, Shanghai, China; ^2^Department of Ophthalmology, Shanghai Sixth People's Hospital Affiliated to Shanghai Jiao Tong University School of Medicine, Shanghai, China; ^3^Department of Computing, The Hong Kong Polytechnic University, Kowloon, Hong Kong SAR, China; ^4^School of Design, The Hong Kong Polytechnic University, Kowloon, Hong Kong SAR, China; ^5^School of Computing, Gachon University, Seongnam, Republic of Korea; ^6^Biomedical and Multimedia Information Technology Research Group, School of Computer Science, The University of Sydney, Sydney, NSW, Australia; ^7^MIRALab, University of Geneva, Geneva, Switzerland; ^8^Shanghai University of Sport, Shanghai, China

**Keywords:** virtual reality, myopia, amblyopia, presbyopia, age-related macular degeneration, Alzheimer, multiple sclerosis, epilepsy

## Abstract

Virtual Reality (VR) has emerged as a new safe and efficient tool for the rehabilitation of many childhood and adulthood illnesses. VR-based therapies have the potential to improve both motor and functional skills in a wide range of age groups through cortical reorganization and the activation of various neuronal connections. Recently, the potential for using serious VR-based games that combine perceptual learning and dichoptic stimulation has been explored for the rehabilitation of ophthalmological and neurological disorders. In ophthalmology, several clinical studies have demonstrated the ability to use VR training to enhance stereopsis, contrast sensitivity, and visual acuity. The use of VR technology provides a significant advantage in training each eye individually without requiring occlusion or penalty. In neurological disorders, the majority of patients undergo recurrent episodes (relapses) of neurological impairment, however, in a few cases (60–80%), the illness progresses over time and becomes chronic, consequential in cumulated motor disability and cognitive deficits. Current research on memory restoration has been spurred by theories about brain plasticity and findings concerning the nervous system's capacity to reconstruct cellular synapses as a result of interaction with enriched environments. Therefore, the use of VR training can play an important role in the improvement of cognitive function and motor disability. Although there are several reviews in the community employing relevant Artificial Intelligence in healthcare, VR has not yet been thoroughly examined in this regard. In this systematic review, we examine the key ideas of VR-based training for prevention and control measurements in ocular diseases such as Myopia, Amblyopia, Presbyopia, and Age-related Macular Degeneration (AMD), and neurological disorders such as Alzheimer, Multiple Sclerosis (MS) Epilepsy and Autism spectrum disorder. This review highlights the fundamentals of VR technologies regarding their clinical research in healthcare. Moreover, these findings will raise community awareness of using VR training and help researchers to learn new techniques to prevent and cure different diseases. We further discuss the current challenges of using VR devices, as well as the future prospects of human training.

## 1. Introduction

Virtual reality (VR) is a technology made up of very interactive computer simulations that track the user's position and replace or augment one or more sense's feedback to create the illusion of immersion or presence in the simulation (1). Impaired vision and the cognitive issues created in daily life can be “experienced” in VR, which is a unique opportunity. Although VR has traditionally been mainly used in the gaming world, it has recently become more widely used in the healthcare sector for medical training and the treatment of a wide range of conditions, including eye diseases, cognitive and anxiety disorders, lowering the risk of accident in older adults, management of pain, obesity-management, loss of concentration during clinical practice, and as a supplement to physical therapy (1–4). However, despite growing awareness of the potential benefits of VR, this innovation is still not sufficiently formed for application in vision impairment and neurorehabilitation.

To ensure that VR interventions and rehabilitation are successful, it is essential to comprehend clinicians' requirements as well as their concerns regarding the effectiveness, usability, and accessibility of VR technology. Among the most important advancements introduced through the development of digital technology, VR has cemented its place in clinical practice, as a new, secure, and efficient method for training, particularly concerning eye and neurological therapies for various conditions that affect children and adults (4). VR enables the users to fully immerse themselves in a simulated world in which they can engage *via* various sensory channels, including visual, aural, cognitive, and haptic, as long as computer-generated 3D environments are presented consistently. Moreover, VR is compatible with high-precision functional imaging methods like functional magnetic resonance imaging (fMRI), researchers can make use of the ability to offer multi-modal stimuli to participants while tracking changes in their brain activity. While their potential benefits for the visual system are largely unexplored, VR-based therapies have been shown to cause cortical reorganization and to promote the activation of different neuronal connections across a wide range of ages (5). As a result, they have significantly contributed to contrasted improvements in some motor and functional skills, such as gait or balance (6).

In practical settings, the majority of researchers who have already deemed the research of VR intervention strategies for neurological and vision disorders have focused on describing the short- and long-term side effects of their use and on recreating various performance tests to attempt to identify psychological and social problems or associated impairments (7), on the other hand, few researchers have only considered the study of VR interventions for neurological and vision disorders (8). These researchers have examined a number of solutions for treating neurological disorders and vision impairment, including the use of various types of commonly produced head-mounted displays (HMDs). Designing a VR game is appropriate for treating up to 95% of diseases. VR may improve a patient's binocular vision, eye-to-eye coordination, cognitive ability, and motor limitations. A good treatment for eye sufferers, according to Khaleghi et al. (9), is to use a VR environment and a filter that dims the patient's vision in their healthy eye and shifts the focus of their vision to the damaged eye. Many experts predict that VR will be the next mass-market platform taking the place of PCs, laptops, and mobile phones. Moreover, clinicians can have significant control over the patient's entire therapeutic experience, which is another advantage of VR.

Therefore, in this systematic review, we investigate the key ideas of VR-based training for prevention and control measurements in ocular diseases such as Myopia, Amblyopia, Presbyopia, and Age-related Macular Degeneration (AMD), and neurological disorders such as Alzheimer, Multiple Sclerosis (MS), Epilepsy, and Autism spectrum disorder which may be treated with the help of serious games that combine sensory training, dichoptic activation, cognitive performance, and motor impairment (10, 11). This combination of technologies enables the doctor to assess, manage, and regulate changes in interocular suppression, one of the causes of cortical abnormalities. In ophthalmology, numerous clinical studies, have demonstrated to enhance stereopsis, contrast sensitivity, and visual acuity (VA). The use of VR technology as a viable treatment for improving vision in eye surgeries has recently gained substantial interest, due to the ability to train each eye separately without the use of occlusion or punishment. One of the principal factors of ophthalmology therapeutic failure, particularly in the pediatric population–may be eliminated by this dichoptic activation strategy due to social stigma, a protracted course of treatment or the physical properties of the patch itself (design, heat, irritation, subpar adhesive material, etc.) (12).

On the other hand, in neurological disorders, the majority of patients undergo recurrent episodes (relapses) of neurological impairment, however, in a few cases (60–80%), the illness progresses over time and becomes chronic, consequential in cumulated motor disability and cognitive deficits. Current research on memory restoration has been spurred by theories about brain plasticity and findings concerning the nervous system's capacity to reconstruct cellular synapses as a result of interaction with enriched environments. Therefore, the use of VR training can play an important role in the improvement of cognitive function and motor disability. Although there are several reviews in the community employing relevant Artificial Intelligence (AI) in healthcare, VR has not yet been thoroughly examined in this regard. The latest innovations in VR training are providing unprecedented options to tackle some major challenges in ocular and neurological diseases. The ability to explain the fundamentals of VR regarding its clinical research will raise community awareness of using VR training and help researchers learn new techniques for studying illnesses.

**Figure 1:**
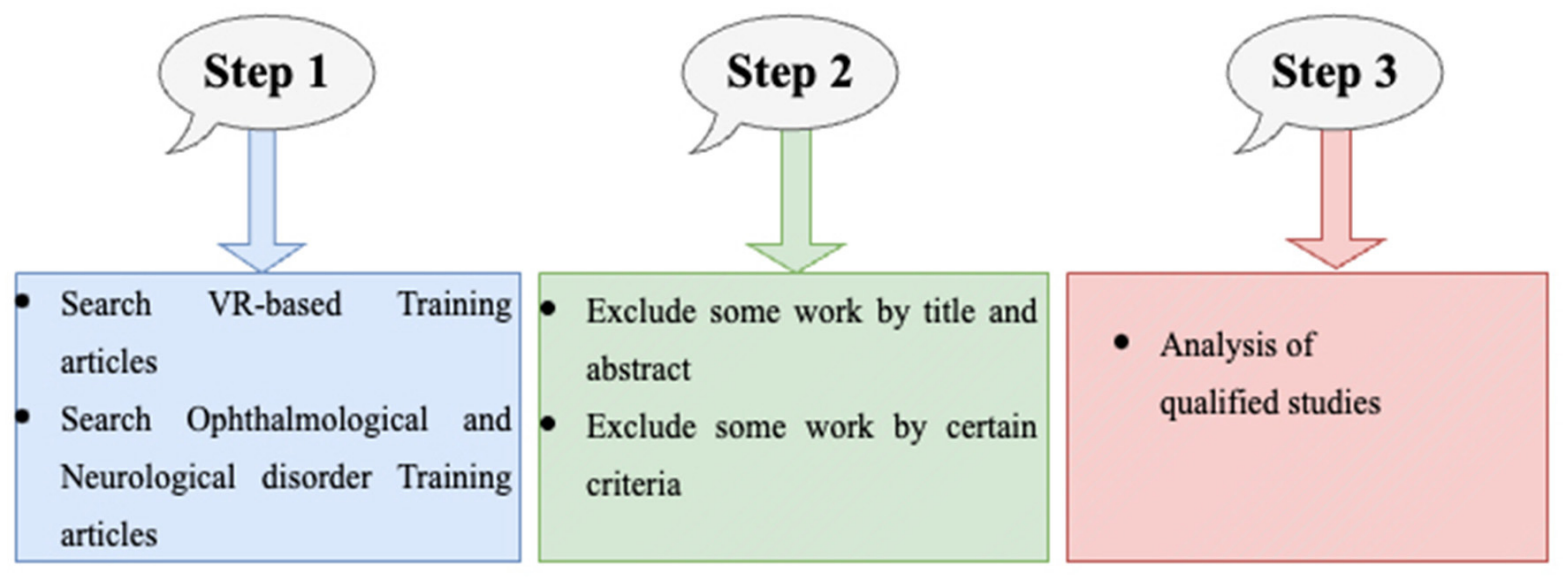
Three steps for database search of systemic review containing VR training.

**Figure 2:**
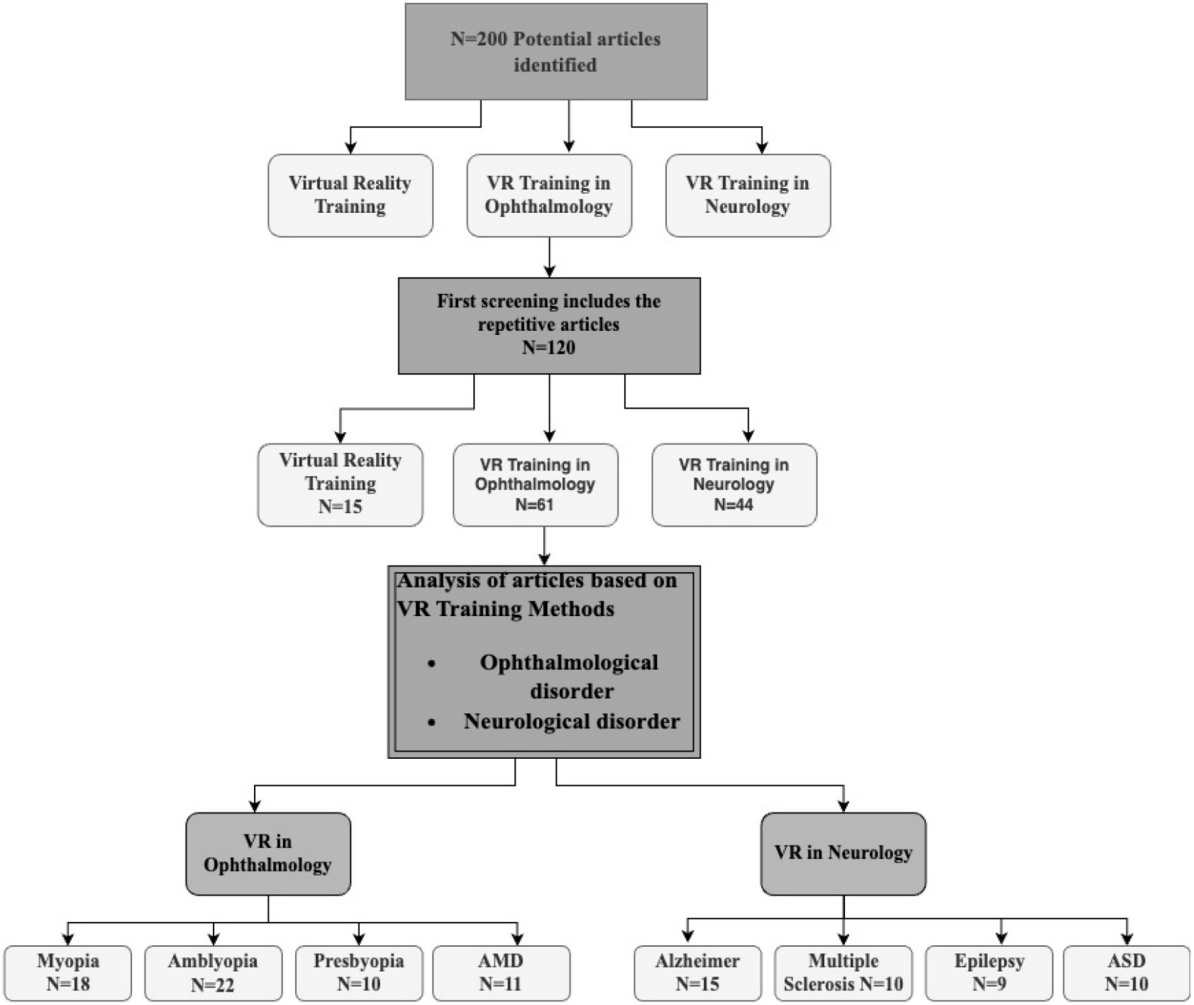
Inclusion and exclusion criteria of citations.

We followed three steps to select the articles (see Figure 1). The first step is to search the VR-based training, VR-based training in opthalmological and neurological disorder articles in databases, and read the titles and abstracts. The second phase involves analyzing the inclusion criteria and excluding publications based on the title and abstract. Analyzing the qualifying works' entire texts is the third and last step. Moreover, in Figure 2, we also explain the literature review citation selection. Initially, we identified 200 articles, afterwards, we include 120 articles based on inclusion criteria.

## 2. Method of literature research

For this overview, we found English articles using the keywords “Virtual Reality,” “Virtual Reality based Training,” and “Prevention and control measurements,” combined with keywords like “Myopia,” “Amblyopia,” “Presbyopia,” “Age-related macular degeneration,” “Alzheimer disease,” “Multiple sclerosis,” “Epilepsy,” and “Autism Spectrum disorder” from the widely used database engines, including Pubmed/MEDLINE, Springerlink, the Cochrane Library, Google Scholar, and EMbase Medline. The collection date is ended in October 2022. The studies that were found using each set of keyword combinations were then amalgamated to create an impartial collection of publications. All references cited in the dataset were thoroughly reviewed by different authors. Proposals protocols, reviews, letters, opinions, studies, and/or articles that were not peer-reviewed were excluded. The references include publications that have been considered because they are pertinent to our topic. In this work, we primarily concentrated on providing a systematic review of VR-based exercises for human ocular, and cognitive diseases. Therefore, we attempt to choose representative VR training that includes preventative and control measures for each category of ophthalmological and neurological disorders covered in this review. In order to provide more of a perspective and opinion review, we admit that not all the articles containing these keyword combinations were included for review.

## 3. VR exercises for prevention and control of human ocular diseases

### 3.1. Myopia

Myopia is caused by the ocular axis' compensatory elongation to the point where it corresponds with the focus when images are focused beyond the retina. One of the most common eye conditions that severely impair vision is myopia. An axial length of >26–27 mm indicates high myopia, which can cause vision loss *via* retinal detachment, neovascularization, cataract, glaucoma, or macular atrophy (13). The axial length of the eye begins to enlarge in childhood and teenage years, with patients between the ages of 8 and 15 experiencing the fastest increase. This prolongation results in axial myopia, a syndrome wherein vision worsens when the axial length of the eye lengthens beyond normal (14).

According to statistics, myopia will affect 49.8% of the world's population by 2050, with a proportion of up to 90% in East Asia and 20% of cases moving to extreme myopia, Pan et al. (15). In America, 33% of adults have myopia, and 4% (16) of those patients have extreme myopia, which is linked to an increased risk of major ocular problems that result in vision loss (17). The greatest global projections for 2020 were made for East Asia (51.6%), and Southeast Asia (46.1%). By 2050, these two regions (East Asia, 65.3%, and Southeast Asia, 62%) will remain responsible for the vast majority of the burden (18). Myopia was also 85–90% common in young adults between the ages of 12 and 18 in Singapore and Taiwan. The age-standardized prevalence of myopia (SE ≤0.75 D) and high myopia (SE <6.0 D) in this age group in Korea was 80.2 and 9.3 respectively, according to a survey of 33,922 patients done from 2008 to 2012 (19).

Myopia is thought to be a multi-factorial disorder with hereditary and environmental variables interacting to cause the condition's rapid rise in prevalence (20). Environmental factors that affect the progression of myopia include things like education and urbanization (21), time spent outside (22), light wavelength (23), and physical activity (24). Adolescent students may experience difficulties in their studies and personal lives as a result of these circumstances, which would have a significant negative socioeconomic impact (25). In order to lower juvenile myopia and avoid excessive myopia, it is imperative to research intervention options for the prevention and control of myopia among teenagers. The main treatment modalities include prescription drugs, vision therapy, glasses, orthokeratology, and laser surgery (26).

**Figure 3:**
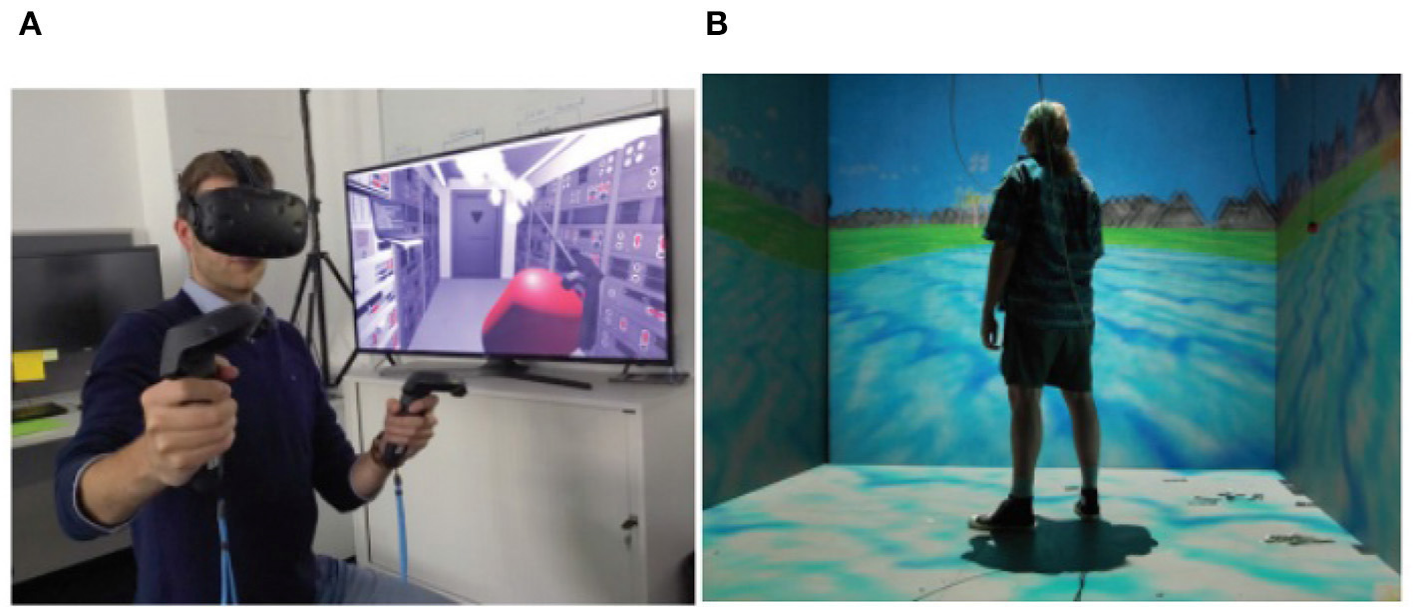
**(A)** HMD iVR By ESA, image is for free distribution under Creative Commons Share Alike License (https://creativecommons.org/licenses/by-sa/3.0-igo). **(B)** CAVE system iVR, copyright free image from Wikimedia Commons (http://commons.wikimedia.org).

The goal of this study is to examine myopia prevention and control methods from the standpoint of visual training. VR-based binocular visual function (BVF) balance training (27), is a form of binocular vision training that patients can receive by wearing VR visual training glasses (Figure 3). In order to avoid myopia and reduce visual fatigue, it helps patients thoroughly exercise their ciliary muscles through visual training, scenes, movies, games, experience halls, and other locations (28). Moreover, these types of training improve movement rate, amplitude, and strength as well as their blood supply and eye focus, all of which helped the subject's eyes accommodate more efficiently without increasing intraocular pressure (IOP). The angle, direction, and visual distance in teenager eyes can be varied in the virtual world while exercising their ciliary muscles all over their bodies as part of the VR-based balance training for BVF has been possible with the aid of HMDs (3). Similarly, Huang et al. (29) discovered that myopia patients' ocular accommodation disorder may be effectively treated with VR-based BVF balancing training.

**Figure 4:**
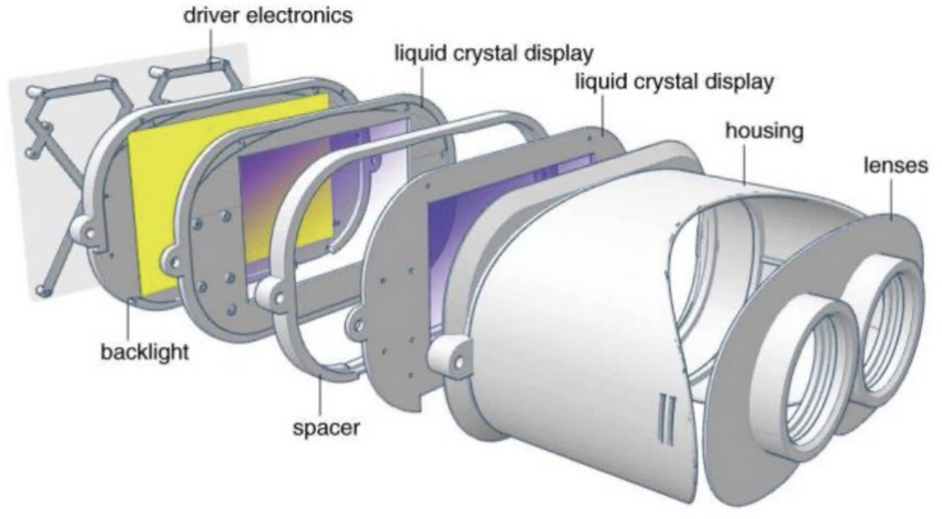
A design of a near eyed light field VR headset (32).

Recently, some companies have introduced VR-based myopia prevention and control devices, claiming that these technologies may effectively treat or even cure juvenile myopia (see Figure 4). One of them is a perfect virtual environment that can be created using VR technology and is unrestricted by time or weather by reproducing various exquisite outdoor scenery and modifying light intensity and spectral composition (28).

The expert found that children need to spend roughly 3 h every day in light conditions that are at least 10,000 lux to be safeguarded against myopia. There are now two established theories explaining how exposure to strong outdoor light can shield against myopia: the ocular axis lengthens and reduces blurring as a result of miosis, which is the first effect of bright exposure to light (particularly defocus-induced blurring). Second, exposure to sunlight can cause the retina to release dopamine, which could help to avoid myopia. However, there are some disadvantage of outdoor exercises like it can be difficult to get 2 h of outdoor exercise every day in northeastern Chinese cities like Beijing and Shijiazhuang in which air pollution may damage children's condition (28), even though numerous studies have demonstrated that enhancing the duration of time invested participating in outdoor recreation tends to help in the prevention and control of myopia. As a result, myopia can be prevented and controlled using VR devices if an immersive outdoor scene with perfect illumination conditions is created, a myopic defocus state is maintained in the patient's peripheral retina through incorporating foveated rendering and eye tracking methods, and accommodation exercise is carried out using specialized software.

### 3.2. Amblyopia

**Figure 5:**
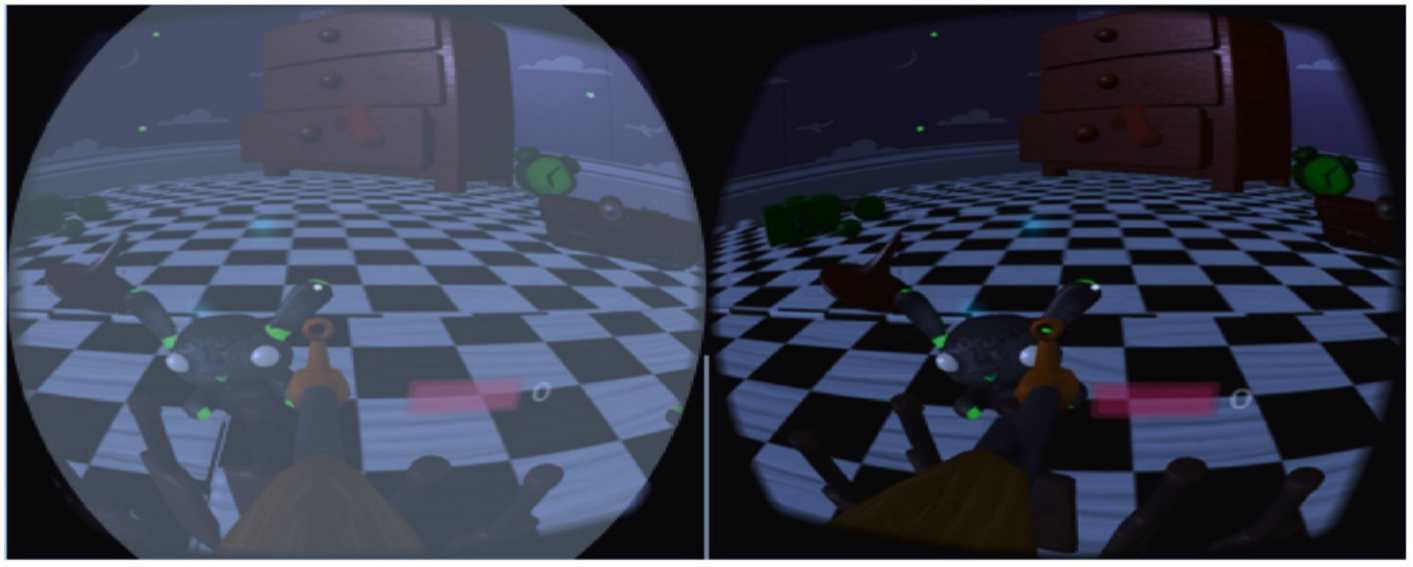
Patient with amblyopia (fellow eye vision is blurry because of a filter used in VR game) (9).

Amblyopia is a type of vision impairment that affects 2–5% of children, and only affects one eye (30) (see Figure 5). The brain starts to ignore the input from the other eye, sometimes referred to as the lazy eye, in amblyopia. As a result, one eye develops stronger whereas the other weakens or becomes lethargic (31). Amblyopia can be caused by four conditions: Strabismus, Refractive or anisometropia, Deprivation, and Mixed. However, strabismus (19%) and anisometropia (50%) are the two leading causes of amblyopia.

Amblyopia can result in issues including social anxiety, poor learning and communication skills, and the inability to participate in group activities, in addition to causing lifelong visual impairment and putting a financial strain on society (33). The geographic location and the level of disease prevalence affect the recurrence of amblyopia in adults. According to Elflein et al. (34), 5.6% of Germans between the ages of 35 and 74 have amblyopia.

Early detection of amblyopia is crucial for prompt treatment of the disorder because it can only be treated while the brain is still developing (35). In any other case, it will persist throughout adulthood, cannot be controlled or treated, and results in either temporary or permanent vision loss (36). Children between the ages of 7 and 13 are seen to react to amblyopia treatment less favorably than children younger than 7. Early detection allows for non-controversial treatment options like patching therapy and wearing spectacles (35). The accurate correction of the refractive error is the initial step in treating amblyopia in adult patients, just as it is with pediatric patients. Many of them started using corrective while they were young and stopped wearing them once they entered adolescence. If the patients wear correction, they rarely wear full anisometric correction. Even in older patients, precise correction improves VA (37). The gold standard for treating amblyopia in children is penalization, which is intended to make them utilize their amblyopic eye (38). Ophthalmologists and the VR applications research team collaborated on a study to look at how VR technology might be used to treat amblyopia. A further advancement in the therapy of amblyopia is playing video games with adult patients, as shown by the beneficial effects of both monocular and binocular training (39). The “compliance” of patients can be greatly aided by motivation in the form of video game play (40). The most recent development in the creation of new pleoptic techniques is the use of VR, which allows for full dichoptic imaging, where each eye sees something slightly different, especially concerning minute details that are essential for controlling the game in question, as well as fundamentally superior 3D imaging. In Halička et al. (41), conducted dichoptic vision therapy using the computer game Diplopia Game (Vivid Vision, USA), which has been played within an Oculus Rift DK2 VR headset (Oculus VR, USA). The workout required 8 h of training every cycle, which was completed twice a week. Each session lasted 60 min and included a variety of games (15–20 min per game). According to comparable trials with young patients, playing a dichoptic video game for 10 h with binoculars improves vision by 1.6 rows (42). The use of anti-suppressive binocular pleoptic training may be more beneficial than monocular pleoptic training for amblyopia, as suppression only manifests physiologically under binocular conditions (43).

Previous cross-sectional studies found that children who play serious games (SGs) have expanded cortical thickness and geographic inventories of gray matter in the dorsolateral prefrontal cortex (PFC), hippocampal formation, frontal eye fields, insula, and cerebellum (44). It has also been clear over time that practicing with SGs leads to improvements in specific visual cognitive skills (45). As an illustration, playing SGs has been proven to increase offers several advantages, including improved contrast sensitivity, improved contrast sensitivity (46), stronger spatial abilities (47), and improved peripheral vision (48). The primary advantage of VR games designed for healthcare is their innate ability to engage players on many different levels (cognitive, physical, perceptual, etc.) and motivate them to keep playing (49). This is especially true for amblyopic children, given their current low compliance with conventional therapy. Similar to this, Qiu et al. (50) presented the Vision-VR^*TM*^ system, a full-field vision system designed to deliver interactive VR video games for binocular amblyopia treatment. The essential component of this technology is the employment of two dissociated optical systems to give unique visual data for each eye, enabling each eye to be activated while wearing specially designed glasses and watching various animated films or participating in interactive VR activities. The patient had a weekly treatment that included 10 min of watching cartoons and 20 min a day of playing VR games as part of the system's initial test.

Another promising method for the treatment of amblyopia is using binocular therapy which trains the patient's eye-to-eye, brain-to-eye, and hand-to-eye coordination through games and visually designed surroundings. As a result, the amblyopic eye improves visual perception and advances stereovision (51). In Waddingham et al. (52), proposed a method *I* − *BiT*^*TM*^, and it was thought that VR might be used to deliver it because it offered control over the images' content, quality, and contrast as well as control over the viewing angle to correct for any eye or interpupillary distance (IPD) deviation. Patients were eager to use the device, and the quick improvement in VA after the first treatment session gave kids and parents an instantaneous favorable response. Moreover, this kind of treatment would not necessitate frequent clinic visits over extended periods as thought previously, and treatment effectiveness appears to occur in under 2 h. Additionally, it was noted (3) that amblyopia patients' best-corrected VA and stereoscopic vision considerably improved after receiving VR-based BVF balance training, indicating the efficacy of this treatment in raising patients' VA levels.

According to all of the available data, VR dichoptic and perceptual learning training appears to be a beneficial therapy alternative for helping amblyopic patients complete their visual rehabilitation. Additionally, these technologies enable medical professionals to monitor and manage variations in interocular suppression, which is thought to be one of the primary causes of cortical abnormalities in amblyopia. More clinical research is required to support the promising outcomes of these treatments, with a focus on defining the best approach for achieving a successful recovery of visual and binocular abilities.

### 3.3. Presbyopia

The accommodation ability is decreased during presbyopia; however, the signal to the extra-ocular muscles (EOMs) and pupils remain. Presbyopia, caused by crystalline lens stiffening, affects about 20% of people worldwide (53). Eye doctors have typically diagnosed presbyopia based on a patient's age or based on clinical tests. Although the symptoms of presbyopia are commonly very predictable in a clinical context, they are much less predictable and very poorly understood when they are experienced in everyday life. Due to the mismatch between accommodation and convergence ability, some people who are experiencing presbyopia may suffer diplopia, or double vision, when staring at close objects. While some patients claim that their eyesight is altered “overnight,” eye professionals are aware that this is a process that begins at a very young age.

By the age of 50, the objective accommodation of an individual is close to zero; however, the subjective accommodation may be higher due to the depth of the pupil's focus (54). Visual performance depends on this arbitrary degree of adaptation. Presbyopia will have a significantly greater influence as the world's demography shifts toward an older population, with at least 40% of the US population being presbyopic (55). The main causes of presbyopia in older people are changes in extra-lenticular characteristics or lenticular alterations (56). Similarly to this, a significant portion of the world's population suffers from various refractive problems in their eyes, necessitating that users wear their prescription glasses in addition to the VR goggles, making the overall experience uncomfortable. VR has the immediate capacity to gather data and offer accurate and dependable training regimens. To reduce the vergence-accommodation conflict, VR and AR systems may use techniques such as dynamic depth of field, which adjusts the focus of the system's lenses in real-time based on the user's gaze, or varifocal displays, which use multiple layers of lenses to simulate the natural accommodation response of the human eye. Additionally, fusional vergence training may be used to help presbyopes effectively diverge to view far-off targets and converge to view close-up objects. For the athlete or driver to acquire the highest accuracy levels of 3D depth perception, several visual abilities are necessary.

**Figure 6:**
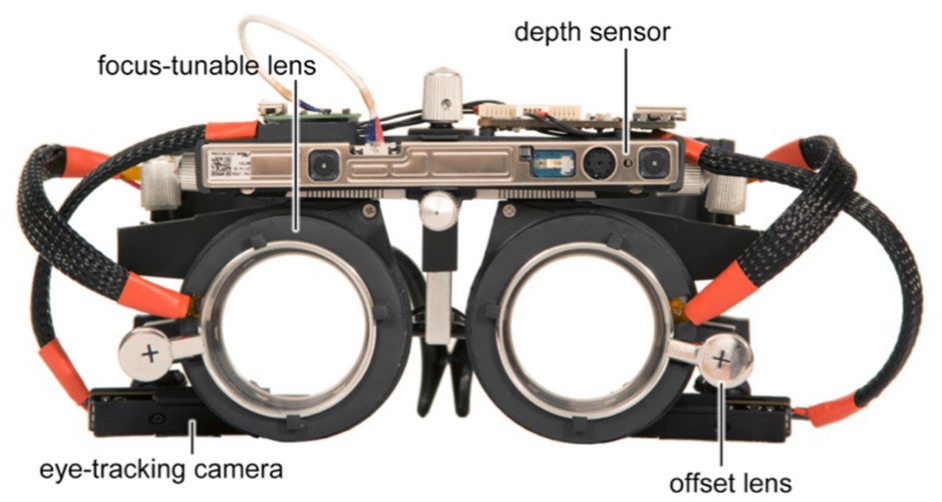
Prototype of presbyopia glasses, this prototype could be a useful tool for both research and clinical applications in the field of vision science and optometry (53).

We assume that autofocals are the first pair of focus-adjustable glasses for correcting presbyopia to have a depth camera and eye trackers, together with a sensor fusion algorithm that combines and efficiently uses both (see Figure 6). It is modeled after gaze-contingent varifocal systems for VR, as mentioned in Padmanaban et al. (57). A VR near-eye light field display (NELD) was demonstrated by Lanman and Luebke (58) building on the integrated imaging ideas that Lippmann had previously presented. Other methods for reducing vergence-accommodation conflict (VAC) in VR NEDs have also been suggested, including mono-vision and the use of focus-tunable lenses (59). By distorting the image displayed, Huang et al. (60) showed that correction for refractive defects may be encoded into a light field display, doing away with the need for glasses. In Chakravarthula et al. (61), depth perception, peripheral awareness, eye tracking, scanning, and visual processing speed are all implemented in a simulated real-space environment to maximize the effect of the training and to create a good transference potential to real-world dynamic environment scenarios (62). It is also assumed that training visual skills in a VR environment could be beneficial, the example images of a VR environment are shown in Figure 3.

### 3.4. Age-related macular degeneration

Age-related macular degeneration (AMD), also referred to as macula lutea, is a common multi-factorial eye disorder of the central part of the ocular posterior segment (63). AMD damages the macular area of the retina, which results in a progressive loss of central vision and has an effect on patients as well as society (64). It is a complicated condition brought on by the interaction of numerous factors (65), including infection (66), and it is the main factor in individuals over 65 who become permanently blind. AMD risk is also known to be increased by injury to the tissues under the macula, Bruch's membrane, or the retinal pigment epithelium. AMD is typically divided into two separate types: the exudative (wet form) and the non-exudative (dry form). According to the literature, AMD affects over 170 million people worldwide and accounts for 9% of all incidents of blindness, making it the third most common cause of blindness globally. According to Wong et al. (67), the prevalence of AMD is expected to increase to 288 million by 2040 as a result of the exponentially aging worldwide population. The average patient's quality of life (QoL) decreased by 17–60% as a result of AMD, which is a considerable burden (68).

People become less independent in completing instrumental and fundamental daily activities, such as walking, feeding, and dressing, as they lose their capacity to distinguish between minute spatial details and their center vision (e.g., taking medications, handling finances, being independent in terms of mobility). This increases their chance of acquiring depression by three times and predisposes them to have low self-esteem (69). Additionally, AMD patients have a doubled risk of falling and a fourfold increased risk of hip fracture (70).

VR-based exercises boost autonomy and enhance the quality of life for those who have lost their eyesight by assisting the patient in adjusting and making the best use of their remaining visual function. A VR program created by the VRMedLab allows users to experience visual impairments from a first-person perspective (71). The viability of using VR approaches in low vision rehabilitation was investigated in this Raphanel et al. (72). One such application makes use of wall-mounted screens that show different cityscapes. They surround the blind patient, which also learns how and where to safely cross the street (2). Another example is eSight, a head-mounted device that is used as an optic aid instead of a rehabilitation tool, aids visually impaired people in navigating daily life situations by improving their perception of the world (8).

There are numerous advantages to VR in particular. First, the patient is aware that the training is simply a simulation, they might feel safer and be more open to trying out other navigational methods (73). Second, the medical professional can further personalize rehabilitation by making it more enjoyable and powerful for the patient. Finally, VR headsets do not require the patient to actively manipulate the program, in contrast to video or computer games that do. Even without a therapist present, therapy sessions can be automated to improve cost-effectiveness and accessibility on a broad scale. A patient can be coached to make the most of his residual visual abilities and learn new visual exploration techniques by mimicking real-world situations in VR. According to Raphanel et al. (72), the patient would proceed to increasingly complicated settings, such as metropolitan streets with moving humans and cars, after becoming comfortable navigating relatively simple simulated environments. Furthermore, some orthoptists worry that patients with AMD who have binocular vision issues will not benefit from a head-mounted device since they lack 3D awareness. This is not true, though, as head-mounted technologies use a hybrid perspective distortion, dynamic stereoscopy, and binocular divergence to produce images. Therefore, a patient can make up for the lack of one of these three facilities by utilizing the other two. Furthermore, employing accelerometers, gyroscopes, and magnetometer sensors, helmet-mounted displays can track the head's position and rotation, ensuring exceptional visual realism and accuracy.

Active visual exploration tactics combining both static and dynamic body movement, combined with hand-eye coordination training (as it can be done with an extra sensor on the patient's hand), may help AMD patients enjoy excellent learning transfer from virtual to real-world situations. These strategies are made possible by VR. Moreover, VR can be adapted to design worlds that are more dynamic and visually complicated for the patient to navigate. To offer standardized low vision rehabilitation to people with AMD, a more effective and economical model has to be built that makes use of evidence-based procedures that have been established by the literature and improved provider training. This VR program provides the chance to develop an enjoyable, immersive environment that enhances learning's real-world application.

## 4. VR exercises for preventing neurological disorders

### 4.1. Alzheimer

Alzheimer's disease (AD) patients mostly have impaired episodic memory functioning, due to deterioration in medial temporal regions (such as the entorhinal cortex, hippocampal formation, and parahippocampal gyrus) (74). According to Foloppe et al. (75), people with AD have trouble completing certain activities (e.g., common place, regularly done tasks, or daily activities (shopping and cooking) and independent life), primarily due to their cognitive impairment.

There were 35.6 million patients of dementia, according to estimation from the World Health Organization (WHO), and this number will be increased to 65.7 million by 2030 and 115.4 million by 2050 (76). The most prevalent pathologies of AD, amyloid plaques and neurofibrillary tangles, have been directly eradicated in phase 3 therapeutic trials, but these efforts have failed to improve clinical outcomes. This suggests that by the time symptoms appear, neuronal death and neuropathology have already had a significant impact on the brain, severely limiting the efficacy of these drugs (77). In this context, treatment administered to pre-clinical patients is of great potential, when prevention may still be possible. Indeed, several significant pharmacologic and non-pharmacologic trials observe people at high risk for AD and also look at how to postpone the onset of cognitive decline.

VR training has the potential to be more interesting and successful, with a high likelihood of transferring the benefits of training to daily life (78). A more dynamic, realistic, and interactive virtual environment can be made by utilizing VR technology (i.e., ecological validity). Notably, both dementia sufferers and healthy older persons have had the feasibility of VR proven (79). In a review of computerized and VR cognitive therapy for participants with mild cognitive impairment (MCI) and dementia, the most coherent advances in the mental abilities of focus, executive function, and brain (visual and verbal), as well as appreciable declines in symptoms of depression and anxiety, were discovered (80). According to the authors' findings, VR and computerized cognitive training are both helpful in halting the progression of cognitive decline (81).

VR training can be viewed as a novel, a sophisticated technique for specifically diagnosing and treating spatial recall impairment (82). Using an HMD and a gamepad, patients can explore a simulated town center, find an object, and memorize its precise location. In virtual environments, a “reorientation job” is easy to implement. More specifically, it is possible to use cognitive training in virtual environments based on particular rehabilitative processes. When paired with passive navigation, Kober et al. (83) offered a fascinating example of how VR can be used to perform a cognitive training program for spatial abilities. A VR-based methodology has also been used to teach the ability to sync between allocentric viewpoint-dependent and allocentric viewpoint-independent representations (84). This demonstrated that VR training also enhanced executive function, opening the possibility to the development of specific protocols for healthy older adults to strengthen the widely used cognitive empowerment strategies for this demographic (85).

**Figure 7:**
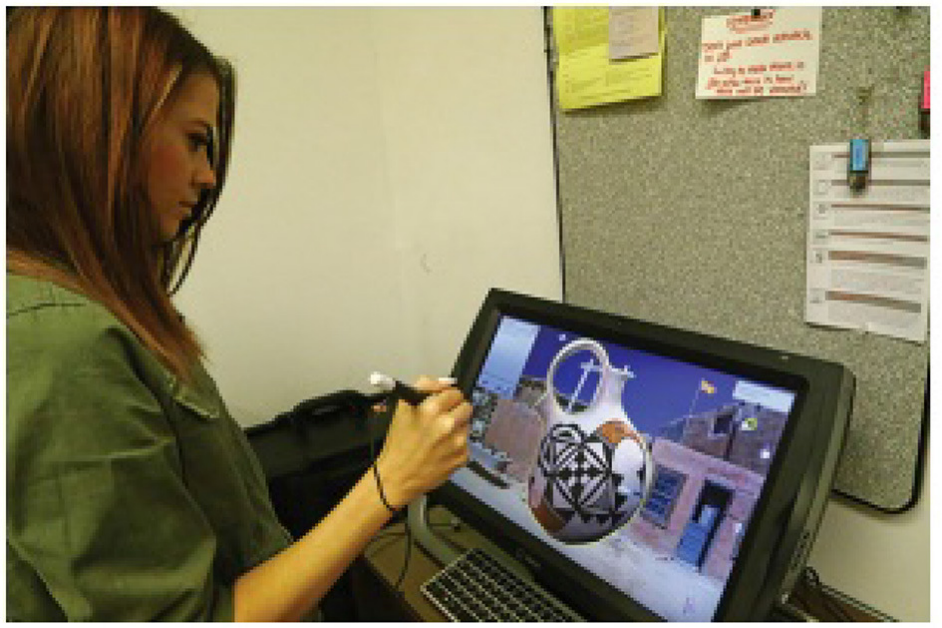
2D virtual environment “Copyright free image from humanities commons: (http://hcommons.org)”.

Indeed, studies have emphasized the significance of AD patients maintaining cognitive activity to stop functional decline over time (86). The development of therapeutic therapies that are likely to maintain or improve their independence in daily activities using techniques that are helpful to them is urgently needed (87). According to previous research (88), a VR-based system is built to help AD patients relearn daily activities. First, VR can offer benefits that are comparable to those observed in real life in the context of rehabilitation (see Figure 7). More importantly, it enables the application of error-less learning and vanishing-cue approaches by offering both a fully controlled environment and automated support systems. Second, studying in a virtual environment can have many extra benefits over learning in a real one. Notably, the learner is continually exposed to a consistent environment in which items are constantly in the same place and there are neither disruptive occurrences nor the presence of objects that are unrelated to the job at hand. Rich and important outcome metrics, including daily activities and navigation, may potentially be an advantage of VR.

### 4.2. Multiple sclerosis

Multiple sclerosis (MS) is known as an inflammatory disease that causes damage to the insulating layer of nerve cells found in the brain and spinal cord (89). According to Compston (90), MS is the second most common cause of neurological disability in adults between the ages of 18 and 50. The disease normally affects persons between the ages of 20 and 50, and women are twice as likely to get MS as men are (89). There are currently 3 million MS patients worldwide. Although there is no known cure for MS, there are several therapies designed to improve functionality after an attack, which stops additional episodes and prevents disability (91).

The physical abilities of MS patients may deteriorate, necessitating human and/or technological support for them to accomplish daily living activities because of the disease's progressive nature, which is highly variable (92). In hospitals and specialized facilities, numerous neurological illness effects are managed on an outpatient basis. Additionally, the majority of MS patients experience mobility, geographic, or a combination of the two challenges that prevent them from obtaining care at a rehabilitation facility. Because of this, there is an increasing interest in developing health initiatives for MS patients' telerehabilitation (93).

In order to increase the patient's motivation, VR provides educational, entertaining, interactive, and immediate feedback. VR is a good representation of the real world (e.g., walking on an uneven or slippery surface, in a busy place, etc). Results from the analyzed studies showed that VR therapy improved MS patients' walking (94), balance (95), arm mobility, and control (96). Additionally, the results demonstrated that VR therapies improved systems for processing and integrating sensory information, enabling anticipatory posture control and reaction mechanisms, and enhancing locomotor characteristics. The authors proposed that VR treatments could serve as both a motivating substitute (93) and an effective therapeutic replacement for traditional motor rehabilitation (89).

VR has been effectively used to enhance the gait performance of the Lokomat (more precisely, the Lokomat-Pro) (97). In contrast to Lokomat-Nanos, Lokomat-Pro offers a feature called augmented performance feedback that enables patients workout functional motions with delight by having their performance evaluated and the results displayed throughout workouts that are tailored to a particular activity. It is possible to adapt this training to the patient's cognitive and motor qualities and receive customized feedback by changing the intensity and level of difficulty of the exercises, which are carried out by the patient's avatar in various virtual environments (98). Furthermore, robotic therapy and VR training sessions are recommended. Researchers found that combining robot-assisted gait training with VR is a more potent therapy approach for MS patients who have problems walking.

A study conducted by Kim et al. (99) found that a 20-min session of walking in a VR environment using the Oculus Rift did not result in any simulator-related illness or adverse effects on postural control, both static and dynamic, in healthy young adults. However, it is important to note that the study was conducted on healthy individuals, and the results may not necessarily apply of a certain age or pathology-related conditions. Additionally, it is possible that prolonged exposure to VR could result in harmful simulator-related consequences in some individuals. Therefore, further research is needed to determine the safety and effectiveness of using VR for clinical purposes, particularly in patients with various medical conditions.

In another study conducted by Canessa et al. (100), the authors compared real walking in a virtual environment with walking in a corresponding real-world situation. The aim of the study was to evaluate the feasibility of using VR in rehabilitation and clinical setups. The study also analyzed the effect of having a virtual representation of the user's body inside the virtual environment. The authors found that there were no significant differences in several spatiotemporal gait parameters between real walking in virtual environments and in real-world situations. These gait parameters included the total distance walked, the patterns of velocity in each considered path, the velocity peaks, the step count, and the step length. Additionally, the authors found that having a virtual representation of the body inside VR did not affect the gait parameters. This study provides evidence that VR can be a feasible and effective tool for gait training in rehabilitation and clinical settings.

In a paper by Nabiyouni and Bowman (101), the authors presented a detailed taxonomy of walking-based locomotion techniques in VR. The taxonomy aimed to provide a framework for better understanding, analyzing, and designing locomotion techniques for VR. The taxonomy consists of six fundamental components of locomotion techniques, including input type, direction, orientation, speed, step size, and synchronization. The authors suggested that designers and researchers could use this taxonomy to create novel locomotion techniques by making choices from the components of this taxonomy, analyzing and improving existing techniques, or performing experiments to evaluate locomotion techniques in detail using the organization mentioned in the article. The taxonomy can serve as a valuable tool for researchers and designers in the field of VR, particularly in the development of locomotion techniques for immersive experiences.

### 4.3. Epilepsy

Epilepsy related to light sensitivity is occasionally referred to as “photoconvulsive” epilepsy, irrespective of the fact that seizures may not be classified as convulsing, therefore, “Photosensitive” is an appropriate modifying term for optical seizures or epilepsy.

The highest prevalence of this illness is seen in the hereditary generalized epilepsies juvenile myoclonic epilepsy (30%-90%), children and juvenile absence epilepsies (18%), generalized tonic-clonic seizures upon awakening (13%) and benign myoclonic epilepsy of infancy (10%) (102). The 17% of juvenile myoclonic epilepsy patients in Japan had visible photosensitivity. Photosensitivity is also a symptom of various other conditions and diseases, including myoclonic epilepsy with ragged-red fibers, type 2 neuronal ceroid lipofuscinosis, Dravet syndrome, Lennox-Gastaut syndrome, Lafora's disease, Unverricht-Lundborg disease, and others Stephani et al. (102).

VR is being utilized more frequently in the medical field for rehabilitation (103), pain management, anxiety reduction (104), and quality of life improvement (105). The impact of VR on seizure tendency has only been studied in a small number of laboratory settings. A prospective study in which patients' game-play during seizures was assessed included patients who were undergoing continuous video/scalp or intracranial electroencephalogram (EEG) monitoring and ictal single-photon emission computed tomography (SPECT) (106). The study sought to evaluate the possibility of a prospective study of driving performance and impaired consciousness during seizures utilizing video games as well as if various types of seizures have varying effects on one's ability to operate a motor vehicle. This type of study would be feasible because driving ability would be negatively impacted by seizure types known to affect consciousness more severely than seizure types not believed to affect consciousness, such as absence, complex partial, and secondarily generalized seizures (e.g., sub-clinical, simple partial).

A number of rodent studies have recently questioned the applicability of VR to understanding the brain's spatial representations (107), whereas others have backed the generalizability of results from VR environments in both rodents and people (108). Many projects [e.g., (109)] have shown the ability to transfer information across real-world and virtual paradigms at the behavioral level. Though it would not be feasible with the majority of epileptic patients at the time, future breakthroughs in the field of mobile VR and AR may allow for such paradigms to be used in future studies [e.g., (108)].

## 5. Autism spectrum disorder

Autism spectrum disorder (ASD) is a neurodevelopmental disorder that affects communication, social interaction, and behavior (110). It is known to be highly heritable and can manifest in a variety of ways, which is why it is referred to as a “spectrum” disorder. The symptoms of autism can range from mild to severe and can co-occur with other conditions such as anxiety, ADHD, and intellectual disability. Early intervention is important for children with autism because it can help them develop communication and social skills, as well as address any challenging behaviors. Parent-mediated interventions and therapist-delivered interventions, such as applied behavior analysis (ABA), are commonly used in childhood. School-based strategies and techniques, such as social skills training and vocational training, can help promote independence and success in adulthood. It's worth noting that while there is no cure for autism, early intervention and ongoing support and therapies can significantly improve outcomes and quality of life for individuals with autism.

Studies have consistently shown that autism is more common in males than females, with a male-to-female ratio ranging from 2:1 to 5:1 (111, 112). The 2010 Global Burden of Disease study estimated the ratio to be 4:1. However, some studies have suggested that the rate of autism in men and women with a moderate-to-profound intellectual disability is equivalent, indicating that the male bias may be influenced by ascertainment bias or diagnostic criteria (112). There is also some evidence to suggest that rates of autism may vary among different racial and ethnic groups (113). Environmental risk factors are believed to play a role in the development of autism. These risk factors could trigger the risk of autism through several complex underlying mechanisms, such as genetic and epigenetic effects, inflammation and oxidative stress, or hypoxic and ischaemic damage (114). However, the exact nature of these risk factors and their interactions with genetic and other factors are still being studied.

VR technologies allow individuals with ASD to actively engage in interactive and captivating situations, which can be used to teach important life skills. VR-based interventions (VRI) have been developed to help individuals with ASD learn and practice skills in a safe and controlled environment. Some examples of VR-based systems that have been developed to teach important life skills to individuals with ASD include driving skills and social skills. Studies have shown that individuals with ASD are able to apprehend, use, and react appropriately to virtual environments and can transfer the skills they learn in VR to real-life situations (115). For example, VR-based social skills training has been shown to improve social communication and interaction skills in individuals with ASD. They can practice social communication and interaction skills in a safe environment and receive immediate feedback from the VR system. VR-based driving simulations have also been used to teach driving skills to individuals with ASD in a safe and controlled environment.

One of the advantages of VR-based interventions is that they can be customized to meet the specific needs and abilities of each individual with ASD, making them a highly flexible and adaptable intervention tool. This customization can include adjusting the level of difficulty of the virtual environment, the type of feedback provided, and the specific skills targeted for intervention. Factors such as age, severity of symptoms, and individual learning styles may impact the effectiveness of VR-based interventions for ASD.

Recent studies have shown that VR interventions can be effective in treating individuals with level 1 ASD, also known as high-functioning autism (HFA) (116). Computer-aided reality simulations, in which individuals with HFA can practice challenging social interactions, have been found to be particularly helpful. Some researchers have focused on using VR interventions to address emotional regulation and social-emotional reciprocity (117), often through the use of avatars or learning games. For example, some studies have used VR to help individuals with ASD identify basic emotions, regulate their emotional expression, and improve their ability to engage in social interactions (118).

A study Frolli et al. (119) examined two types of interventions for enhancing social skills: i) emotional training achieved through VR and (ii) conventional emotional training conducted alone with a therapist. For the suggested social tasks, they sought to determine the intervention with the quickest acquisition time. According to their findings, the acquisition time for main emotion recognition was the same for both intervention modalities. The group using VR, on the other hand, had quicker acquisition times for both primary and secondary emotions. This suggests that VR-based interventions may have some advantages over traditional interventions in certain areas of social skills training for individuals with ASD. Overall, VR-based interventions have the potential to be a valuable tool for individuals with ASD to learn and practice important life skills in a safe and controlled environment. However, more research is needed to determine the effectiveness of these interventions and to identify which individuals with ASD may benefit most from them.

## 6. Interaction between immersive VR environments

The development of interactive and collaborative virtual environments that may be used to create powerful digital games has been made possible by the expansion of immersive VR technologies, such as low-cost HMDs. Here, we have reviewed some articles that provide a collaborative environment prototype that allows users to interact with objects while submerged in VR. In Gusai et al. (120), designed a basic exercise game where the player is required to grab and move things. The scene is visualized using the HTC Vive HMD, and the author used two different modalities to interact with it: one modality makes use of the controllers that the HTC Vive offers, and the other modality makes use of the Leap Motion, a cheap hand tracker designed to provide a natural interaction in VR. This study's objective was to identify which of the two devices provides the best alternative for usage in the context of a manipulation task, both in terms of performance on the task as a whole and in terms of the experience of the people involved.

According to another paper (121), experiments were carried out utilizing high levels of interaction fidelity (IF) in VR to enhance user experience and immersion, however, there is evidence that low IF can produce results that are just as good. In a subsequent study, they investigated how users perceived IF for whole-body movements in a VR game that let users crawl under boulders and “dangle” from monkey bars. According to their research, high IF is preferred for manipulating objects, while moderate IF can be sufficient for whole-body movements due to a trade-off between usability and social considerations.

Developers and designers of VR applications aim to evoke a sense of presence, the fundamental sensation of “being” or “acting” in a synthetic world even while the user's own body is physically located somewhere else. Several studies have discovered that the degree of avatar realism and the number of limbs affect the sense of presence in VR. In Kocur et al. (122), research with 24 individuals revealed that the absence of the index fingers in particular decreases presence results in the highest phantom pain ratings, and drastically alters hand-interaction behavior. The relative employment of the thumb and index fingers, as opposed to the middle, ring, and little fingers, was likewise found to be higher with abstract hands than with realistic ones, even when the fingers were absent.

## 7. Conclusion and future directions

The evaluated studies have revealed positive findings and potential benefits of using VR technologies for exercise as a beneficial alternative in the process of visual and neurological rehabilitation. VR exercises can be more engaging and motivating than traditional rehabilitation exercises, which may lead to increased participation and adherence to the treatment plan. Another benefit of VR-based exercises is that they can be conducted in a safe and controlled environment, which can help patients feel more comfortable and confident during the rehabilitation process. Furthermore, VR technologies can provide more immersive and realistic sensory stimulation, which can be particularly beneficial for patients with visual and neurological impairments. It can also allow the creation of customized exercises that can be tailored to the specific needs and abilities of each patient, which can improve the effectiveness of treatment. Moreover, VR technologies can provide objective measurements of patients' progress and performance, which can help clinicians track their recovery and adjust their treatment plans accordingly. Overall, VR technologies have shown promising results in improving the effectiveness of visual and neurological rehabilitation exercises.

However, it is important to carefully evaluate the results, and additional research is necessary to overcome limitations such as small sample sizes and the absence of control groups. The identified VR-related factors such as excessive stress, game difficulty, high physical demands, spasticity, and sensory loss should be taken into consideration by doctors in the rehabilitation exercise process and should be clinically assessed. In addition, the cost and analysis of the evaluation strategy should also be considered, and potential biases in the employed technologies should be addressed.

While the results indicate the potential benefits of employing VR for rehabilitation, further research is needed to improve the effectiveness of treatment for patients with neurological and visual disorders. By continuing to investigate the potential of VR technologies in rehabilitation, we can develop better strategies to help patients achieve their recovery goals.

## Data availability statement

The original contributions presented in the study are included in the article/supplementary material, further inquiries can be directed to the corresponding author.

## Author contributions

SA and XW: conceptualization. SA, PL, YJ, and BS: writing. LB, JK, YC, DF, NM, and JW: review and editing. The article's submission was reviewed and approved by all authors.
